# PTEN loss promotes oncogenic function of STMN1 via PI3K/AKT pathway in lung cancer

**DOI:** 10.1038/s41598-021-93815-3

**Published:** 2021-07-12

**Authors:** Guangsu Xun, Wei Hu, Bing Li

**Affiliations:** Department of Thoracic Surgery, The First Affiliated Hospital of Zhengzhou University, No. 1. Eastern Jianshe Road, Zhengzhou, 450052 Henan China

**Keywords:** Cancer, Oncology

## Abstract

Among all cancer types, lung cancer has already become the leading cause of cancer-related death around the world. The molecular mechanism understanding this development is still needed to be improved to treat lung cancer. Stathmin (STMN1) was initially identified as a cytoplasmic protein phosphorylated responding to cell signal and controlled cell physiological processes. The dysregulation of STMN1 is found in various kinds of tumors. However, the molecular mechanism of STMN1 regulating lung cancer is still unclear. Here, we found that STMN1 was overexpressed in lung cancer tissues and associated with worse survival rates of lung cancer patients. Inhibition of STMN1 suppressed lung cancer cell growth, migration and invasion, and promoted drug sensitivity. Moreover, PTEN loss promoted STMN1 expression via PI3K/AKT pathway. PTEN loss ameliorated the inhibition of cell growth, migration and invasion, and drug sensitivity induced by STMN1 knockdown in lung cancer. The high expression of STMN1 was negatively correlated with the low expression of PTEN in lung cancer specimens. Overall, our work demonstrated that PTEN regulated the oncogenic function of STMN1 in lung cancer.

Among all cancer types, lung cancer has already become the leading cause of cancer-related death around the world^1^. Non-small cell lung cancer takes up the most kinds of lung cancer patients, more than 80%^2^. Despite new therapy options to emerge including targeted therapy and immune checkpoint inhibitor, the mean survival rates of lung cancer patients remain too low^3,4^. Following a large number of reports to investigate the lung cancer pathologic progression, the molecular mechanism understanding this development is still needed to be improved^5,6^.

Dysregulation of various genes has been implicated in the initiation and progression of cancer^7–9^. The overexpression of stathmin (STMN1) is found in all sorts of tumors and related to tumor growth, metastasis and poor survival^10–12^. Given the oncogene function of STMN1, STMN1 is also called as oncoprotein 18^13^. These findings suggested that STMN1 could play a crucial role in tumorigenesis and development^14,15^. In human cervical cancer, STMN1 participated in arsenic trioxide- (As_2_O_3_)-induced apoptosis. Increased expression of STMN1 benefitted anchorage-independent cancer cell growth by cJun. However, the molecular mechanism of STMN1 regulating lung cancer is still unclear.

PTEN (phosphatase and tensin homolog deleted on chromosome 10) as a notable tumor suppressor possesses both protein and lipid phosphatase activity^16^. PTEN's important role in tumor is widely investigated. And the PI3K/AKT pathway is commonly activated in cancerigenesis and development^17^. PTEN conducts by negatively regulating PTEN/PI3K/Akt signaling pathway that regulates multiple cellular functions such as cell growth, apoptosis and migration.

Here we investigated the function of STMN1 gene in lung cancer and might molecular mechanism underlying lung cancer progression. We found that overexpression of STMN1 changed lung cancer cell growth, migration, invasion and drug sensitivity. PTEN loss in lung cancer regulated the expression and function of STMN1.

## Results

### Overexpression of STMN1 in lung cancer

**Figure 1:**
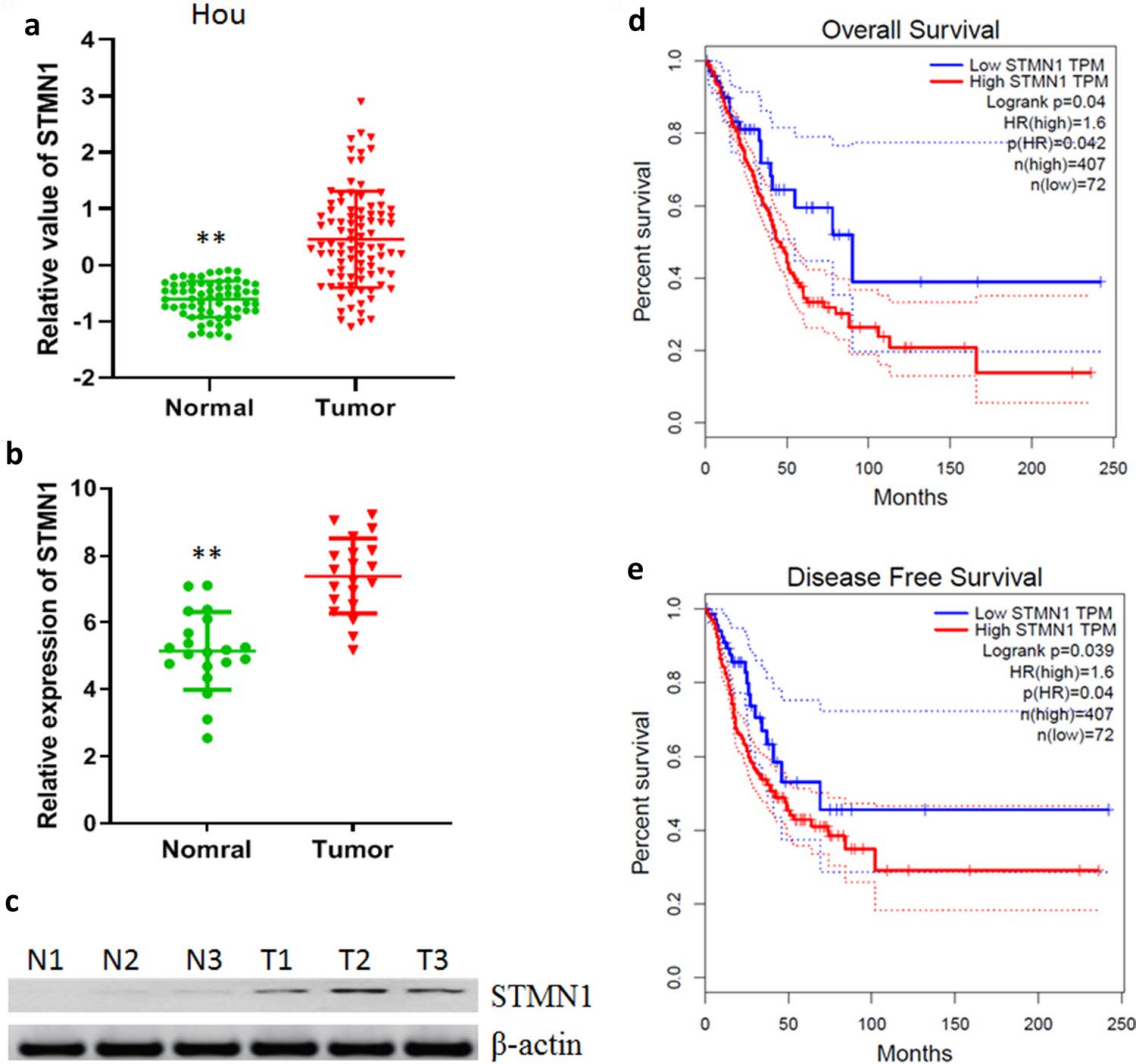
Overexpression of STMN1 in lung cancer. (**a**) The expression of STMN1 in Hou datasets. **p < 0.01. (**b**) The expression of STMN1 in lung cancer tissues and adjacent normal tissues was detected by real time PCR. **p < 0.01. (**c**) The expression of STMN1 in lung cancer tissues (T) and adjacent normal tissues (N) was detected by western blotting. Full-length images are presented in Supplementary Fig. 1. (**d**,**e**) Kaplan–Meier curve of the overall and disease-free survival rates in lung cancer patients with low and high expression of STMN1 (*p* = 0.04).

To identify the potential role of STMN1 in lung cancer, the clinical relevance of STMN1 expression in lung cancer was firstly evaluated. We retrieved public TCGA datasets from Hou cohort and assessed STMN1 gene expression in lung cancer^18^. We found that the expression of STMN1 in lung cancer was higher than normal tissues (Fig. 1a). Here, the expression of STMN1 was evaluated in our data by real time PCR, and the results showed that STMN1 expression was also up-regulated compared with normal tissues (Fig. 1b). Next, we evaluated the protein of STMN1 in lung cancer by western blotting and found that STMN1 protein level was higher than normal tissues (Fig. 1c). Finally, survival rates in lung cancer patients were analyzed from GEPIA (http://gepia.cancer-pku.cn/). We found that lung cancer patients with high STMN1 expression had worse overall and disease-free survival rates, respectively (Fig. 1d,e). Taken together, these results suggested that STMN1 was overexpressed in lung cancer tissues and associated with worse survival rates of lung cancer patients.

### Inhibition of STMN1 suppressed the cell growth, migration and invasion of lung cancer

**Figure 2:**
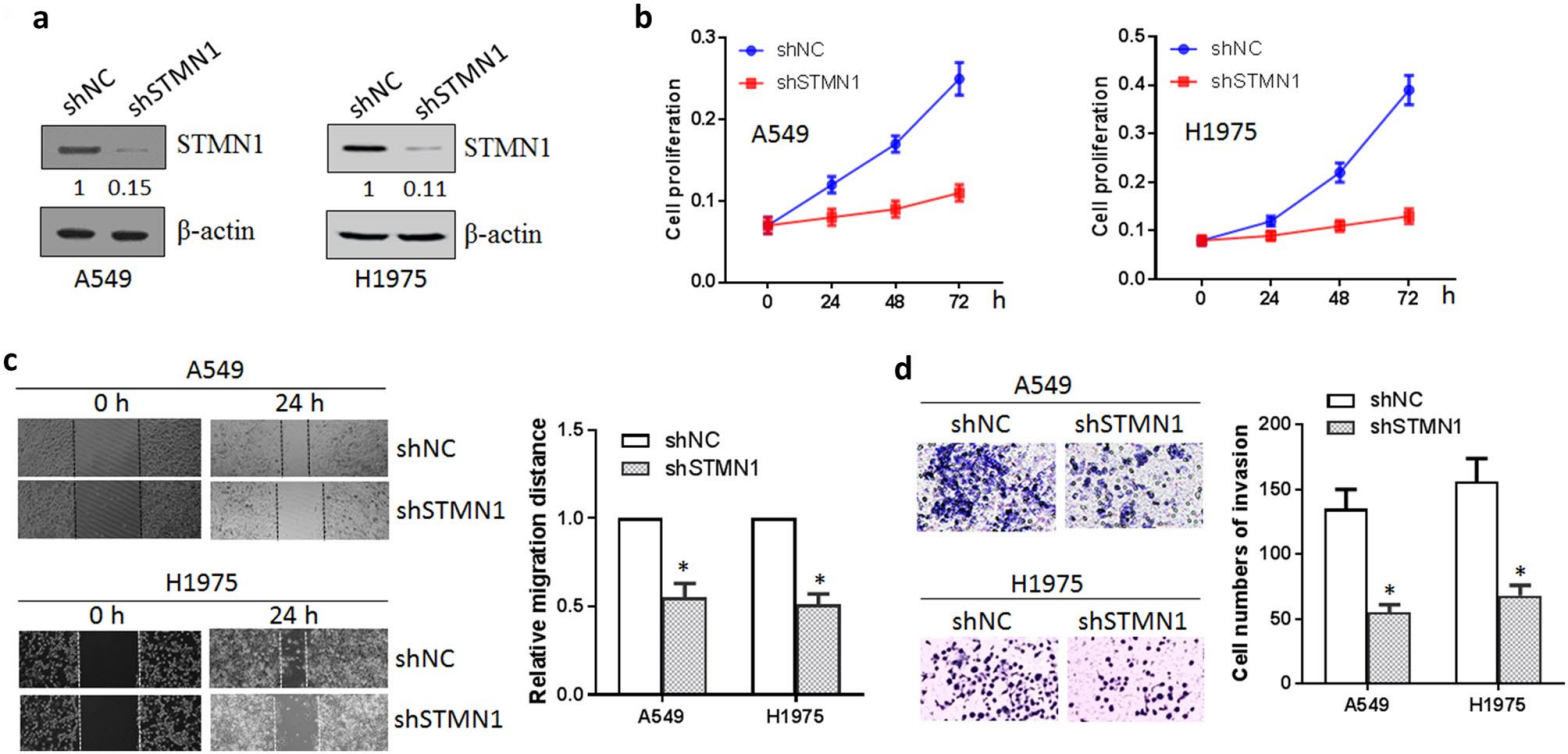
Inhibition of STMN1 suppressed the growth, migration and invasion of lung cancer cells. (**a**) Knockdown of SMN1 in A549 and H1975 cells was measured by western blotting. Full-length images are presented in Supplementary Fig. 2. (**b**) Cell proliferation was decreased after knockdown of STMN1 in A549 and H1975 cells. (**c**) Silencing of STMN1 inhibited the migration ability of A549 and H1975 cells. *p < 0.05. (**d**) Silencing of STMN1 also inhibited A549 and H1975 cells invasion. *p < 0.05.

To investigate the biological function of STMN1 in lung cancer, STMN1 shRNA and control was transfected into lung cancer cell line A549 and H1975^19^. Western blotting confirmed the down-regulated STMN1 expression (Fig. 2a). Firstly, cell proliferation was conducted by cell counting kit-8 (CCK-8) assay, and we found that knockdown of STMN1 markedly suppressed the cell viability of lung cancer (Fig. 2b). To assess cell migration and invasion ability, wound healing and transwell assay was performed. The results showed that silencing of STMN1 significantly inhibited lung cancer cell migration compared with control group (Fig. 2c). Consistent with migration assay results, knockdown of STMN1 also inhibited lung cell invasion ability (Fig. 2d). These suggested that STMN1 as an oncogene was involved in lung cancer progression by regulating cell proliferation, migration and invasion.

### Knockdown of STMN1 led to the sensitivity to paclitaxel

**Figure 3:**
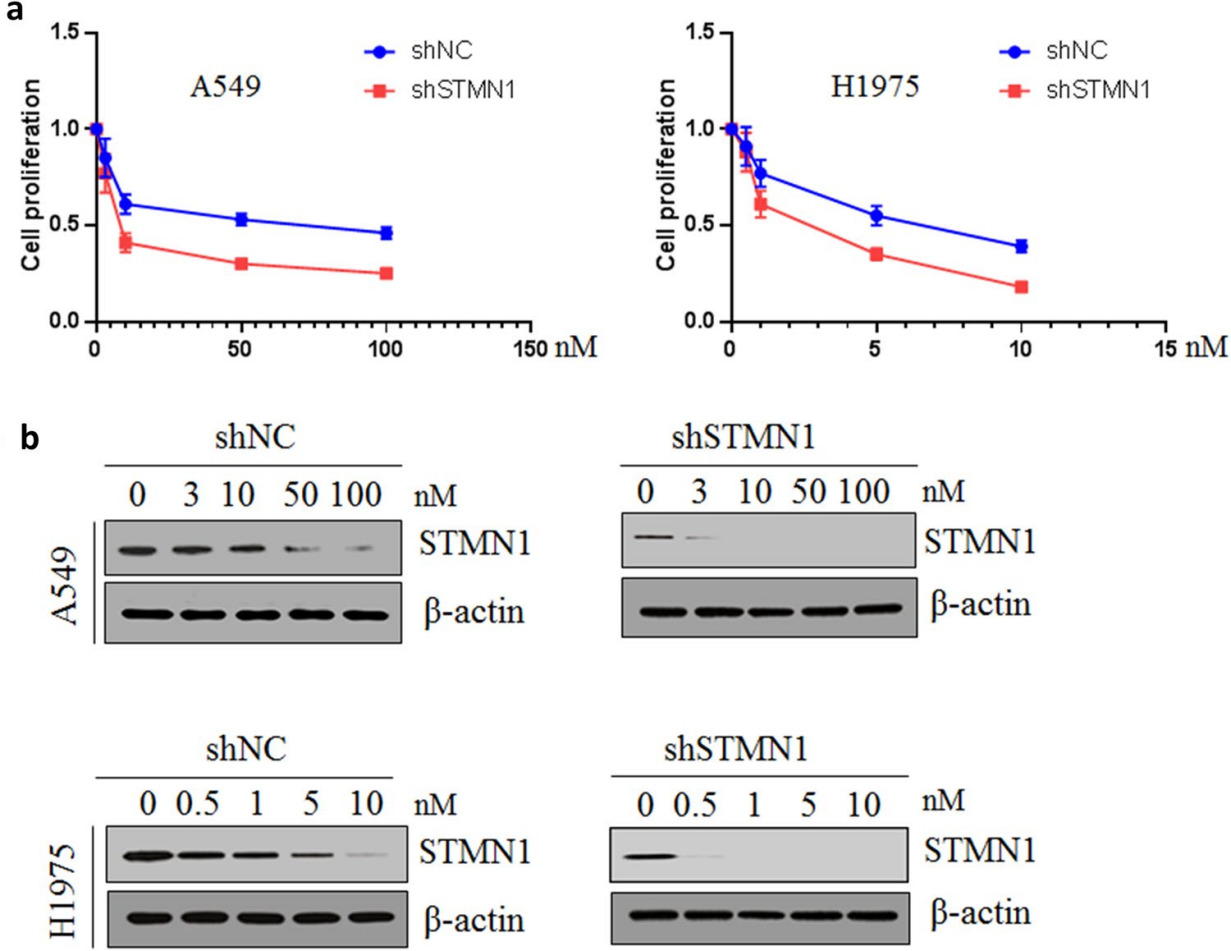
Knockdown of STMN1 led to the sensitivity to paclitaxel. (**a**) Lung cancer cell A549 and H1975 was treated with different concentration of paclitaxel, and cell proliferation was evaluated after knockdown of STMN1 in lung cancer cells. (**b**) Western blotting was used to detect the expression of STMN1 after lung cancer cell A549 and H1975 was treated with different concentration of paclitaxel. Full-length images are presented in Supplementary Fig. 3.

Next, we assessed the effect of STMN1 on sensitivity of lung cancer cells to cancer drug. The growth of lung cancer cell A549 and H1975 with 10^4^ cells/mL plated on a 96-well plate being treated with paclitaxel for 24 h being treated with paclitaxel in different concentrations was determined by CCK-8. Our results showed that the knockdown of STMN1 enhanced the sensitivity to paclitaxel of A549 and H1975 cells (Fig. 3a). The expression level of STMN1 protein was also studied after lung cancer cells were treated with paclitaxel, and western blotting showed that paclitaxel decreased the STMN1 expression level (Fig. 3b). These results suggested that knockdown of STMN1 sensitized lung cancer cells to paclitaxel.

### PTEN loss promoted STMN1 expression via PI3K/AKT pathway

**Figure 4:**
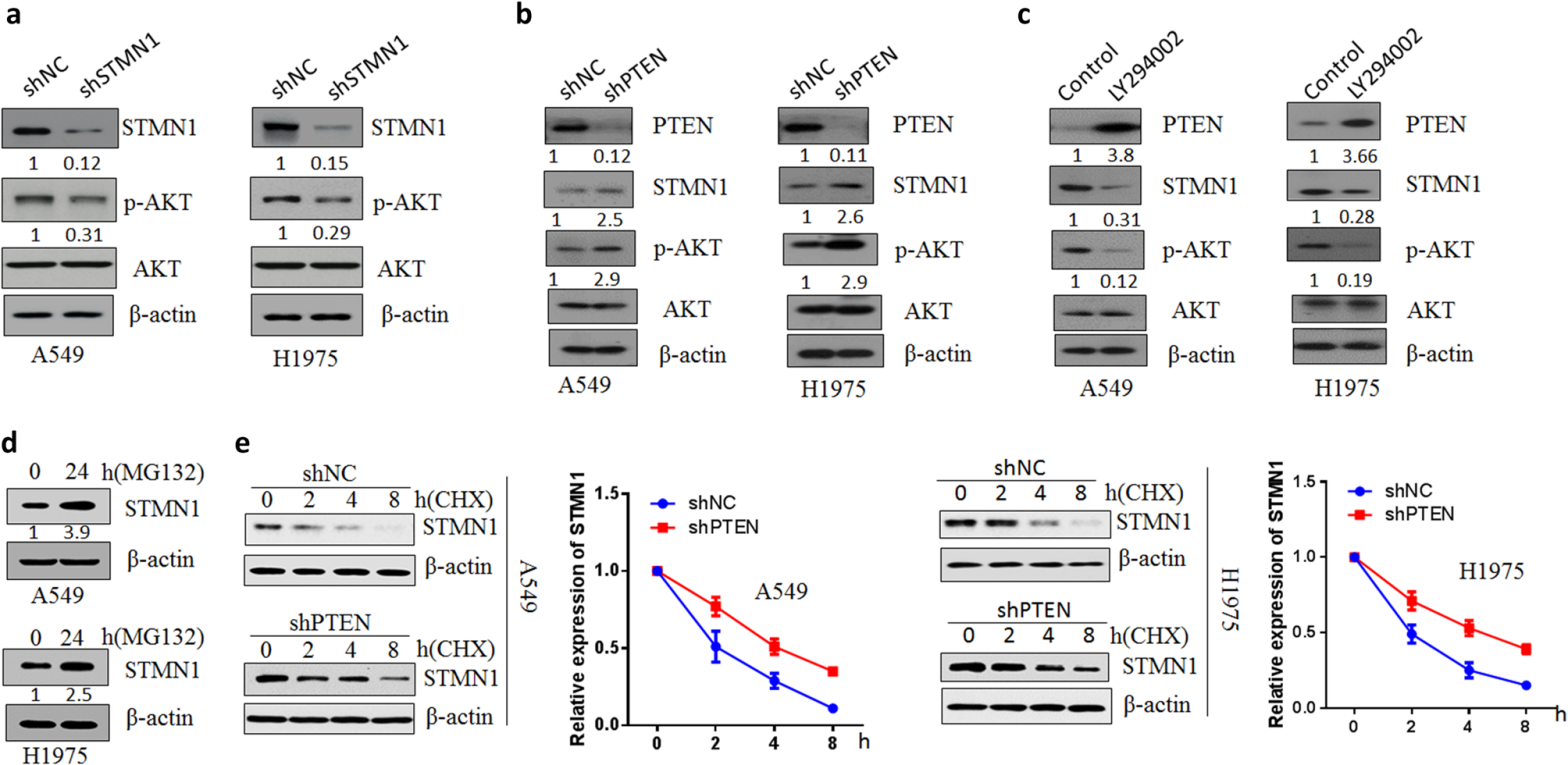
PTEN loss promoted STMN1 expression via PI3K/AKT pathway. (**a**) Western blotting analysis of STMN1, p-AKT and AKT protein levels in A549 and H1975 cells transfected with STMN1 shRNA and negative control plasmids. Full-length images are presented in Supplementary Fig. 4. (**b**) Protein levels of PTEN, STMN1, p-AKT and AKT were detected via western blotting in A549 and H1975 cells transfected with PTEN shRNA and negative control plasmids. Full-length images are presented in Supplementary Fig. 5. (**c**) Western blotting analysis of PTEN, STMN1, p-AKT and AKT protein levels in A549 and H1975 cells treated with PI3K-kinase inhibitor LY294002. Full-length images are presented in Supplementary Fig. 6. (**d**) A549 and H1975 cells were treated with MG132. Full-length images are presented in Supplementary Fig. 7. (**e**) A549 and H1975 cells with or without PTEN knockdown were treated with CHX (cycloheximide, CHX) (10 µg/mL). Western blotting was used to analyze the protein level of STMN1. Full-length images are presented in Supplementary Fig. 8.

It is reported that STMN1 expression has been associated with PI3K-mediated signal transduction pathway^20^. To investigate the mechanisms of STMN1 regulating lung cancer cell progression, we detected PI3K/AKT signaling pathway. As Fig. 4a shown, knockdown of STMN1 decreased the p-AKT protein activity. We further study the role of suppressor gene PTEN in the mechanisms of STMN1 function in lung cancer. Inhibition of PTEN expression in lung cancer cells promoted the expression of STMN1 and p-AKT (Thr-308) protein activity (Fig. 4b). When lung cancer cell A549 and H1975 were treated with PI3K-kinase inhibitor LY294002 for 48 h, the results demonstrated that LY294002 increased the expression of PTEN and decreased the STMN1 and p-AKT protein levels (Fig. 4c). Moreover, when STMN1were knocked down and synergized with LY294002 treatment in A549 and H1975 cells, the combination significantly inhibited cell proliferation (Supplementary Fig. 9). Since PTEN loss enhanced STMN1 protein level, we wondered if PTEN affected the stability of STMN1 protein. As Fig. 4d, the use of proteasome inhibitor MG132 promoted the expression of STMN1 and decreased the degradation of STMN1. Flowingly, PTEN loss promoted the stability of STMN1 after lung cancer cells were treated with CHX (cycloheximide, CHX) (Fig. 4e). Furthermore, MG132 treatment together with knockdown of PTEN in lung cancer cells make STMN1 protein more stable (Supplementary Fig. 10). These suggested that PTEN loss promoted STMN1 expression via PI3K/AKT pathway.

### Effect of PTEN on the progression of lung cancer cells induced by STMN1

**Figure 5:**
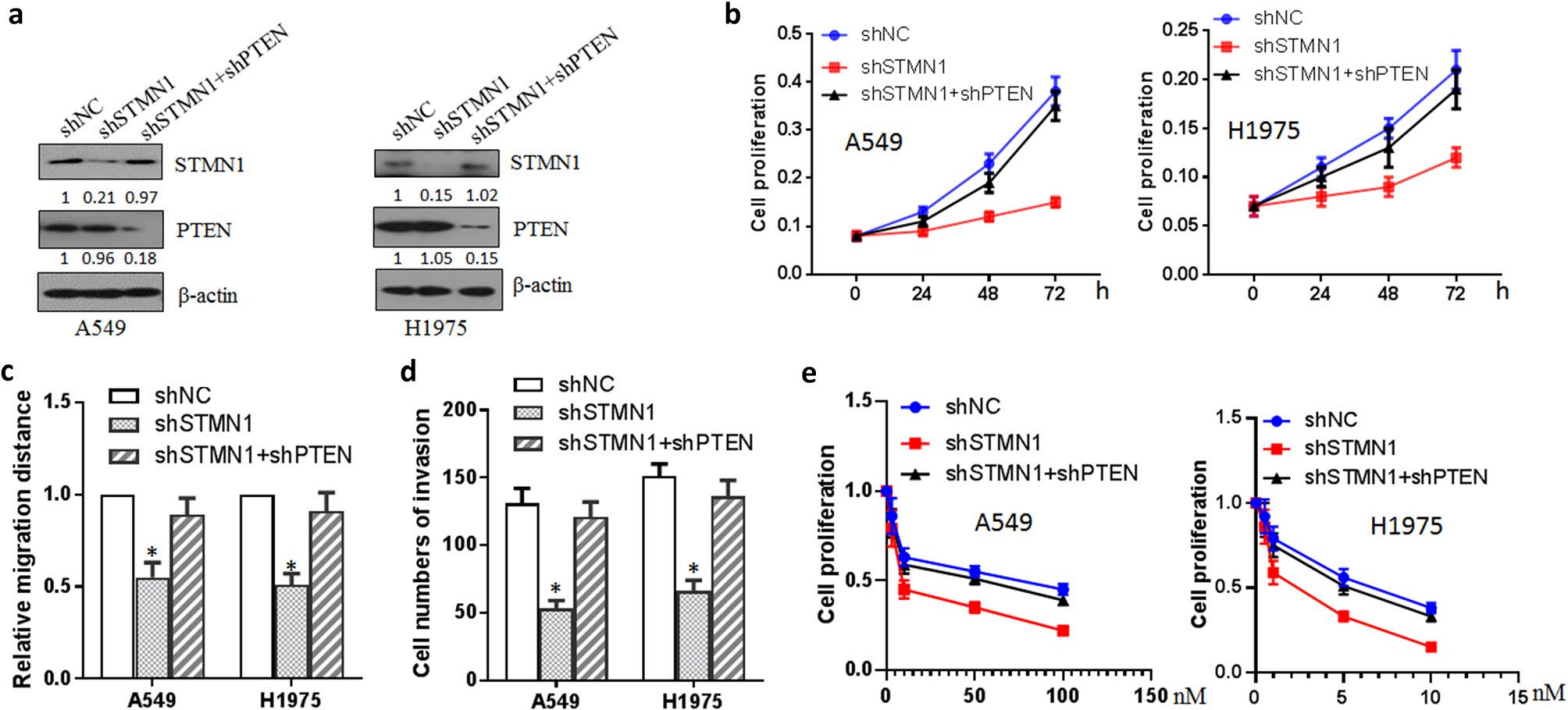
Effect of PTEN on the progression of lung cancer cells induced by STMN1. (**a**) Western blotting analysis of STMN1 in lung cancer cell A549 and H1975 transfected with STMN1 shRNA with or without PTEN shRNA plasmids. Full-length images are presented in Supplementary Fig. 11. (**b**) Cell proliferation was analyzed by CCK-8 kit in lung cancer cell A549 and H1975 transfected with the indicated plasmids. (**c**) Cell migration was analyzed by wound healing in lung cancer cell A549 and H1975 transfected with the indicated plasmids. *p < 0.05. (**d**) Cell invasion was analyzed by transwell in lung cancer cell A549 and H1975 transfected with the indicated plasmids. *p < 0.05. (**e**) Cell proliferation was analyzed by CCK-8 kit to determine the sensitivity of lung cancer cell A549 and H1975 to paclitaxel.

The effect of PTEN on STMN1 inducing lung cancer cell growth was further investigated. Western blotting showed that knockdown of PTEN rescued the expression of STMN1 protein level in A549 and H1975 cells (Fig. 5a). Cell proliferation analysis of lung cancer illustrated that knockdown of PTEN ameliorated the effect of STMN1 knockdown inducing decreased cell proliferation when double RNA interferences of STMN1 and PTEN were performed (Fig. 5b). Wound healing assay showed that double knockdown of STMN1 and PTEN raised lung cancer cell migration compared with single knockdown of STMN1 (Fig. 5c). Transwell assay also showed that knockdown of PTEN ameliorated the effect of decreased cell invasion ability induced by STMN1 knockdown (Fig. 5d). Cell proliferation was also used to determine the sensitivity of lung cancer cell A549 and H1975 to paclitaxel. As Fig. 5e shown, knockdown of PTEN ameliorated the effect of STMN1 knockdown inducing high sensitivity to paclitaxel of lung cancer cell.

### Expression of STMN1 and PTEN in lung cancer specimens

**Figure 6:**
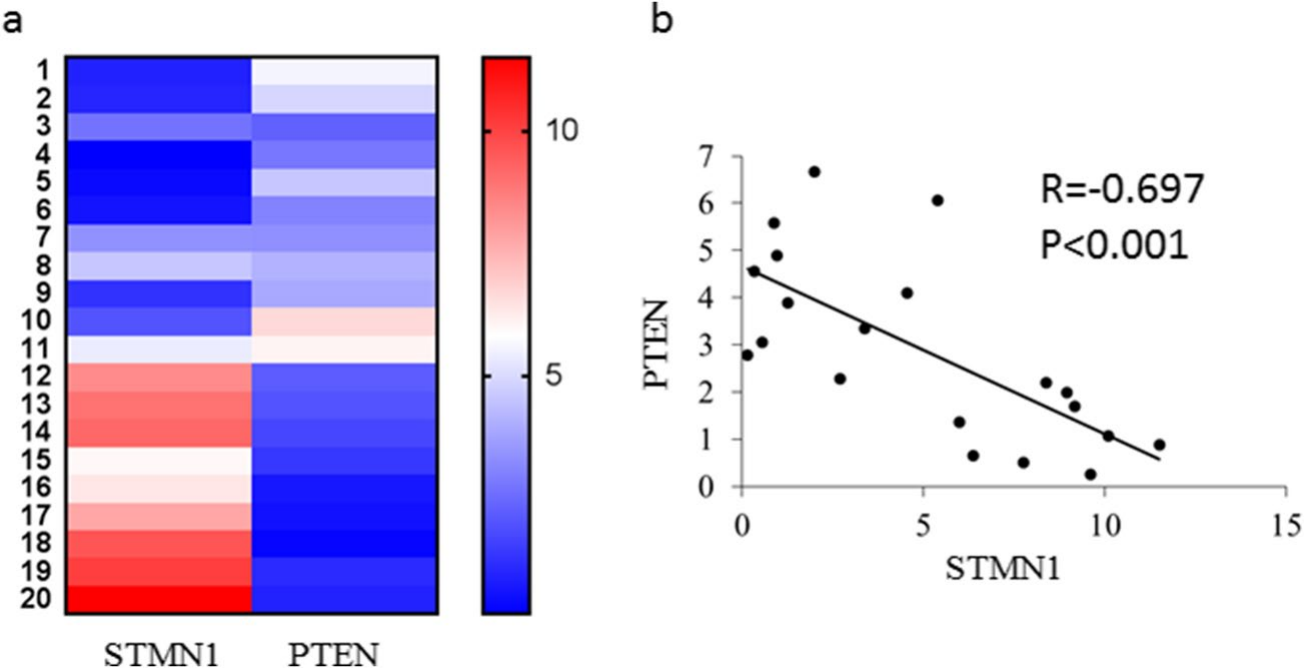
Expression of STMN1 and PTEN in lung cancer specimens. (**a**) Real time PCR was used to detect the mRNA level of STM1 and PTEN in lung cancer specimens. (**b**) The correlation between STMN1 and PTEN expression level in lung cancer specimens was analyzed.

The STMN1 and PTEN mRNA expression levels in lung cancer specimens were detected by real time PCR. Our results showed that STMN1 expression was significantly up-regulated in lung cancer specimens compared with normal ones, however, PTEN expression was down-regulated in lung cancer specimens (Fig. 6a). The correlation of STMN1 with PTEN was assessed and we found that there was a negative correlation between STMN1 and PTEN expression in lung cancer specimens (Fig. 6b).

## Discussion

STMN1 was initially identified as a cytoplasmic protein phosphorylated responding to cell signal, such as growth factors^21^. STMN1 is also a key regulator of microtubule dynamics to destabilize microtubules by binding tubulin dimers^22–24^. STMN1 is involved in multiple diseases to play a pivotal role in cellular processes. As an oncogene in cancer cell biology, STMN1 functions in various cancers progression including cell proliferation, differentiation and cell cycle. Consistent with these data, we found that STMN1 was overexpressed in lung cancer tissues and associated with worse survival rates of lung cancer patients. It was reported that STMN1 were higher levels in pancreatic cancer than in the corresponding normal tissues^25,26^. STMN1 was highly expressed in hepatocellular carcinoma and closely associated with shorter survival time in hepatocellular carcinoma patients^27,28^. We observed that the inhibition of STMN1 suppressed lung cancer cell growth, migration and invasion and promoted drug sensitivity. Similarly, knockdown of STMN1 in prostate cancer resulted in reduced proliferation and invasion of cells, tumor growth and metastasis^29^. In prostate cancer, exposure of cancer cells transfected with an anti-stathmin adenovirus to Taxol or etoposide induced a complete inhibition of proliferation and clonogenicity, and a notably increase in apoptosis^30^. In gastric cancer, STMN1 expression is related to cancer curability, recurrence, and resistance to adjuvant therapy. The knockdown of STMN1 inhibited gastric cancer cells proliferation and sensitized the cells to paclitaxel^31^.

The mechanisms of STMN1 function in cancers are also studied. In prostate cancer, prostate-derived Ets transcription factor (PDEF) down-regulated oncogenic STMN1 expression to inhibit prostate cancer progression at transcription level^32^. Karyopherin-α2 regulated STMN1 by import of E2F1/TFDP1 in liver cancer^33^. In colorectal cancer miR-193b directly targets STMN1 to inhibit the malignant phenotype^34^.

Next, we also investigate the molecular mechanism regulating STMN1 and found that PTEN gene affected the expression and function of STMN1 in lung cancer. PTEN as a known tumor suppressor, whose loss is widely observed in both heritable and sporadic cancers^35^. Various studies have focused on the clinical pathological significance of PTEN in cancer progression and confirm its tumor-suppressive role in multiple tumor types including lung cancer^36,37^. PTEN protein loss is observed to be a frequent event in lung cancer and undertakes an anti-oncogenic duty^38^. Likewise, our study validated the anti-tumor role of PTEN in lung cancer. We found that PTEN loss promoted the expression level of STMN1 and knockdown of PTEN ameliorated the effect of STMN1 inhibition inducing the suppression of lung cancer cell function. PTEN can conduct by PI3K/AKT pathway in various cancers^39,40^. The PTEN/PI3K/AKT pathway regulates multiple cancer cell functions, such as cell growth, proliferation and invasion. PTEN/PI3K/AKT pathway alterations was also found in NSCLC^41^. PTEN as a phosphatase combats the oncogenic function of PI3K/AKT pathway. In present study, PTEN loss promoted STMN1 expression via PI3K/AKT pathway. Our results were similar with the above reports. Here, as the inhibition of PI3K/AKT pathway affected the expression of STMN1 and the knockdown of STMN1 also regulated the activity of PI3K/AKT pathway, the STMN1 and PI3K/AKT pathway might form a feedback loop to function. Moreover, we observed that the high expression of STMN1 was negatively correlated with the low expression of PTEN in lung cancer specimens. However, there are some limitations in this study. Given the effect of PTEN knockdown on STMN1 function, we think that it is necessary to further investigate the function of STMN1 in PTEN/null cells and animals. So that whether the mechanisms of PTEN function is available in all cancer types or whether inhibiting STMN1 is a viable target for all cancer subtypes needs to be further explored. Maybe STMN1 treatment should be avoided in PTEN null cancers.

In conclusion, this study demonstrated that overexpression of STMN1 changed lung cancer cell growth, migration, invasion and drug sensitivity. PTEN loss in lung cancer regulated the expression and function of STMN1.

## Methods

### Cell culture

A549 Cells were cultured in DMEM (Dulbecco’s modified Eagle’s medium) and H1975 cells were cultured in 1640 medium (Gibco) both containing 10% (v/v) fetal bovine serum (Gibco) at 37 °C in a 5% CO_2_ cell culture incubator.

### Clinical specimens

All patients diagnosed with lung cancer were obtained in the First Affiliated Hospital of Zhengzhou University from August 2015 to August 2019. Lung cancer tissues and the adjacent normal tissues from patients were frozen in liquid nitrogen after resection. All the patients have not received any chemotherapy or radiation treatment before surgery. All patients provided written informed consent and this study was approved by the Ethics Committee at the First Affiliated Hospital of Zhengzhou University and were performed in accordance with the relevant guidelines and regulations.

### Real-time PCR

The total RNA from tissues or cells was isolated using TRIzol reagent (Invitrogen) according to the instruction. RNA concentration and purity was determined before RNA was reverse transcribed into cDNA with RT primers using Reverse Transcription kit (Applied Biosystems). Gene expression was measured by ABI 7500 Fast Real-Time PCR System. Relative gene expression was calculated by 2^−ΔΔCT^ method.

### Western blotting

Cells or tissues was collected using Trypsin–EDTA solution and lysed with RIPA lysis buffer. After the protein concentrations were measured, each cell lysate was separated by 10% sodium dodecyl sulfatepolyacrylamide gel electrophoresis and transferred to polyvinylidene fluoride membranes (Millipore). The membranes were incubated with the corresponding primary antibody and then secondary antibody. The proteins were visualized with enhanced chemiluminescence (Millipore) according to the manufacturer’s instructions. The blots were cropped prior to hybridisation with antibodies to save reagents etc.

### Cell proliferation assay

Cell proliferation was examined with a CCK-8 kit (Dojindo Molecular Technologies) according to manufacture protocol. Simply, cells were plated in 96-well plates. At the corresponding time, after 10 μL CCK-8 reagent was added into each well, the plate was then incubated at 37 °C for 2 h. The absorbance at 450 nm was examined with a reader.

### Cell migration and invasion assay

A wound healing assay was performed to analyze cell migration. The wound of lung cancer cells was created by a sterile pipette tip and continued incubating in medium for 24 h. The migrated distance of lung cancer cells under a microscope was captured. The relative migrated distance of cells is measured by the distance of cell migration/the distance measured at 0 h.

The Invasion assays was measured with a transwell system using 8 μm chambers (Corning). The transwell chambers were coated with 250 μg/mL BD Matrigel. Simply, 200 μL cells at a density of 2 × 10^5^ cells/mL in FBS-free medium were then seeded into the top chambers, and 600 μL complete medium was added to the bottom chambers. After incubation at 37 °C for 48 h, the upper cells of transwell inserts were scraped and the lower invasive cells were stained with crystal violet and counted under a microscope.

### Statistical analysis

Data were analyzed using GraphPad Prism 8 (GraphPad software). Data were presented as mean ± standard deviation. All results were obtained at least 3 independent experiments. Student’s t-test was used for the comparison of two group data, and one-way ANOVA was used for the comparison among multiple group data (≥ 3). Spearman’s correlation was used to test the significance of association between genes. The difference analysis with a two-tailed *p* < 0.05 was statistically significant.

## Supplementary Information

Supplementary Figures.
