# Supplementary material for: PTEN loss promotes oncogenic function of STMN1 via PI3K/AKT pathway in lung cancer
Source: Sci Rep. 2021 Jul 12;11:14318. doi: 10.1038/s41598-021-93815-3 (PMC8275769; doi:10.1038/s41598-021-93815-3)

**PTEN loss promotes oncogenic function of STMN1 via PI3K/AKT pathway in lung cancer**

Guangsu Xun^*^, Wei Hu, Bing Li

Department of Thoracic Surgery, The First Affiliated Hospital of Zhengzhou University, Zhengzhou, Henan, China.

Running title: PTEN loss promotes STMN1 function

***Corresponding author**. Department of Thoracic Surgery, The First Affiliated Hospital of Zhengzhou University, No.1.Eastern Jianshe Road, Zhengzhou, Henan 450052, China.

Email: xungsu@163.com

Supplementary figure 1. Original blot data of figure 1c.


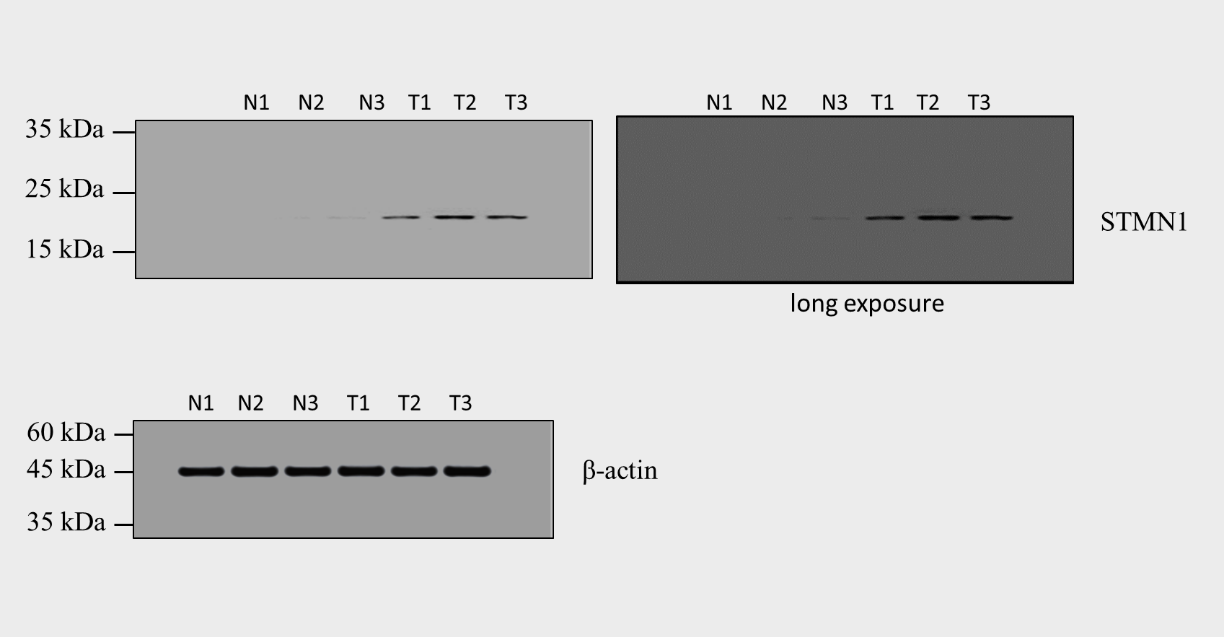


Supplementary figure 2. Original blot data of figure 2a.


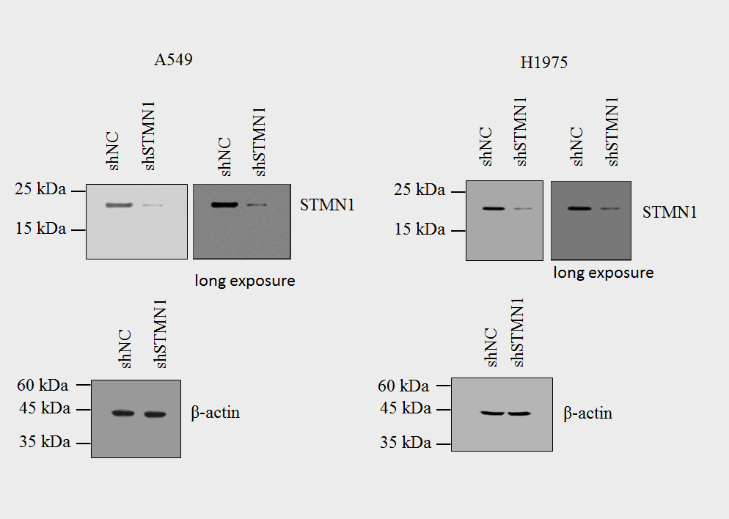


Supplementary figure 3. Original blot data of figure 3b.


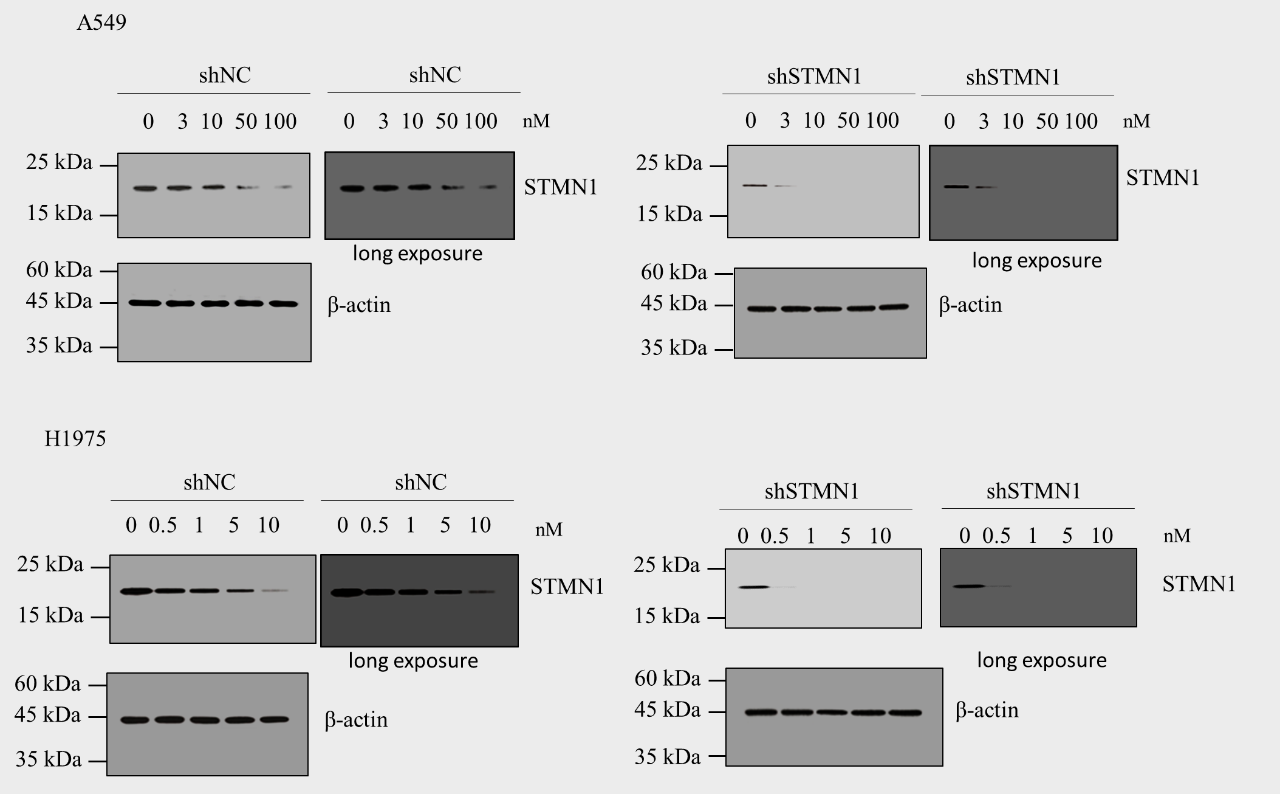


Supplementary figure 4. Original blot data of figure 4a.


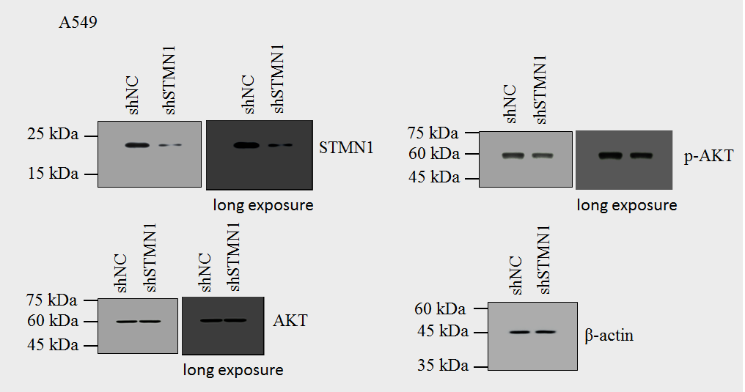


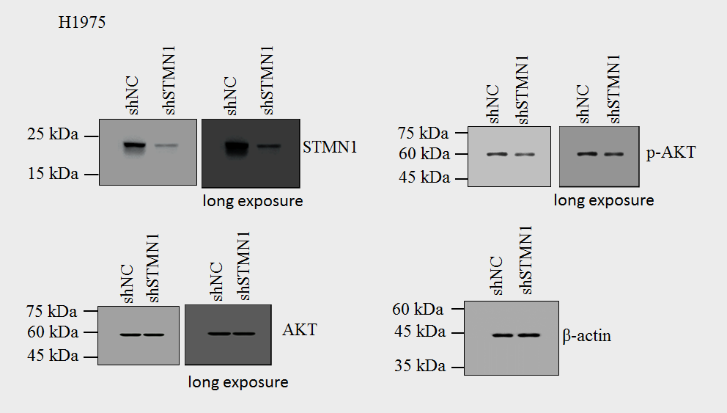


Supplementary figure 5. Original blot data of figure 4b.


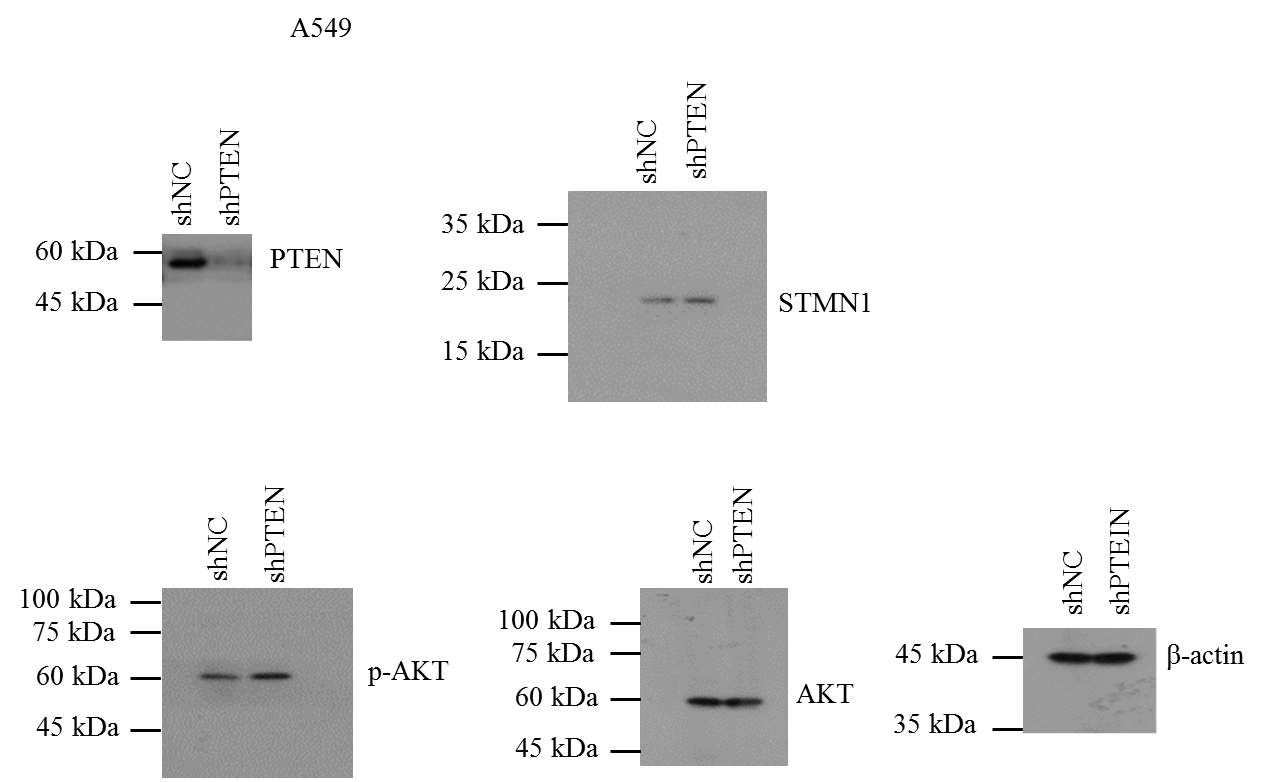


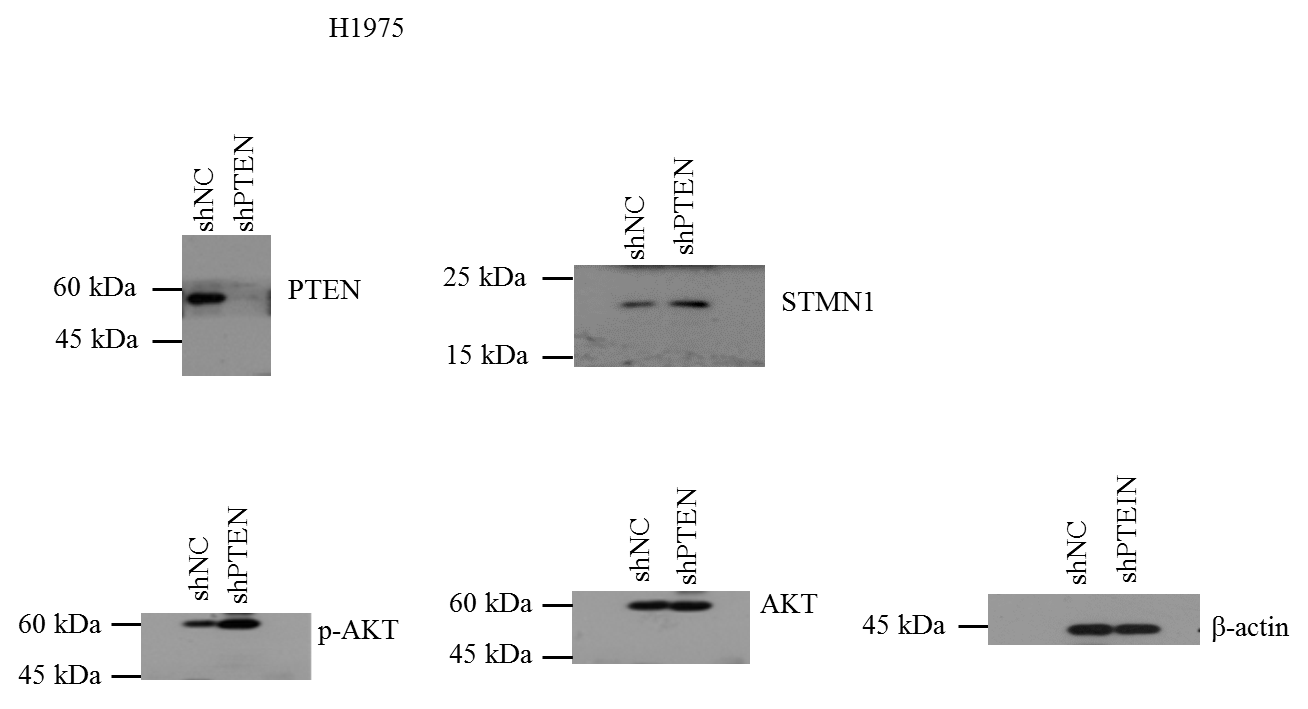


Supplementary figure 6. Original blot data of figure 4c.


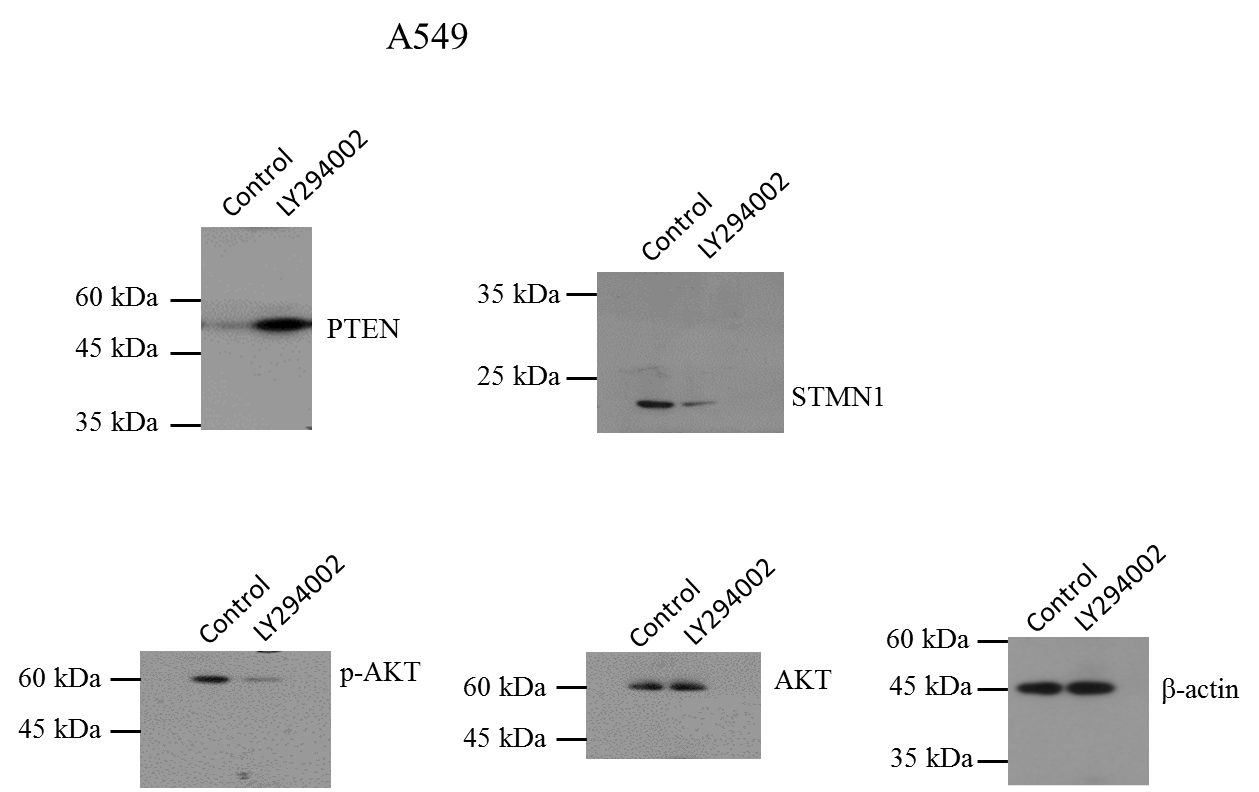


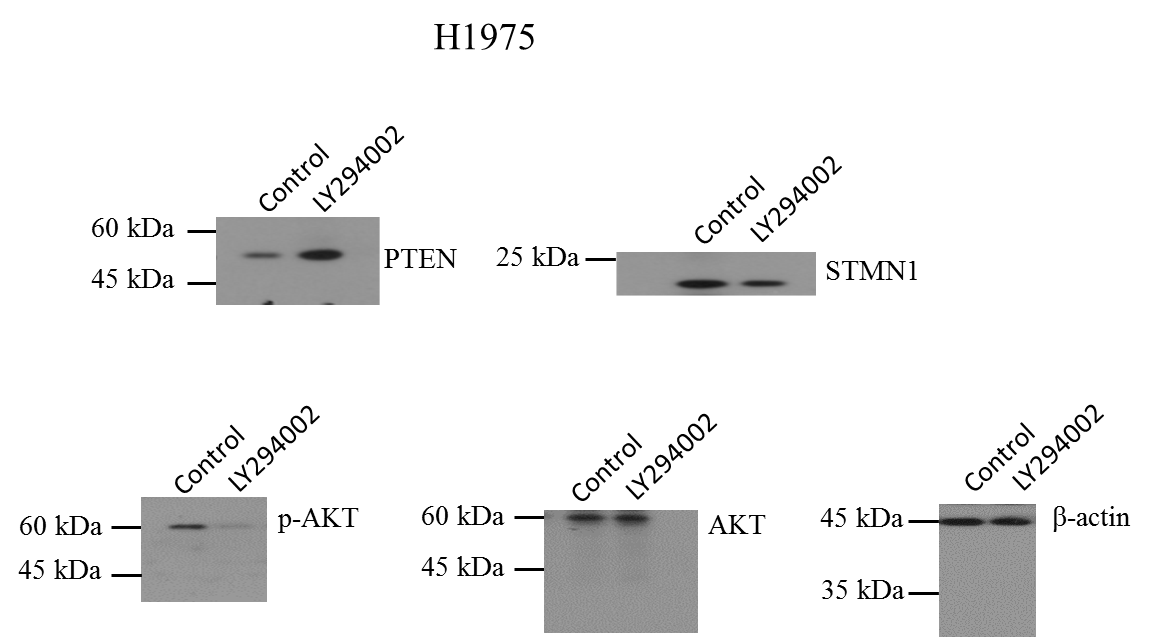


Supplementary figure 7. Original blot data of figure 4d.


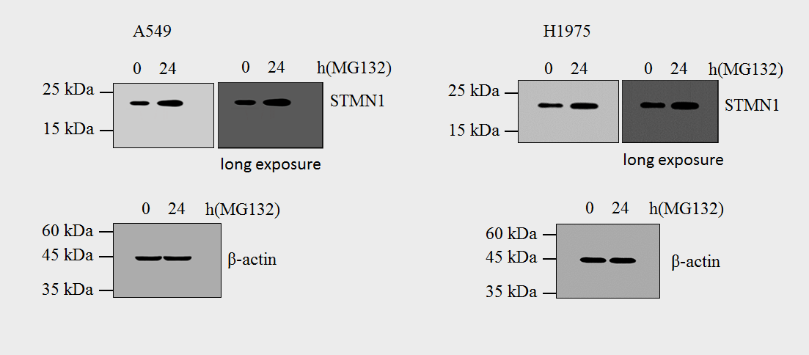


Supplementary figure 8. Original blot data of figure 4e.


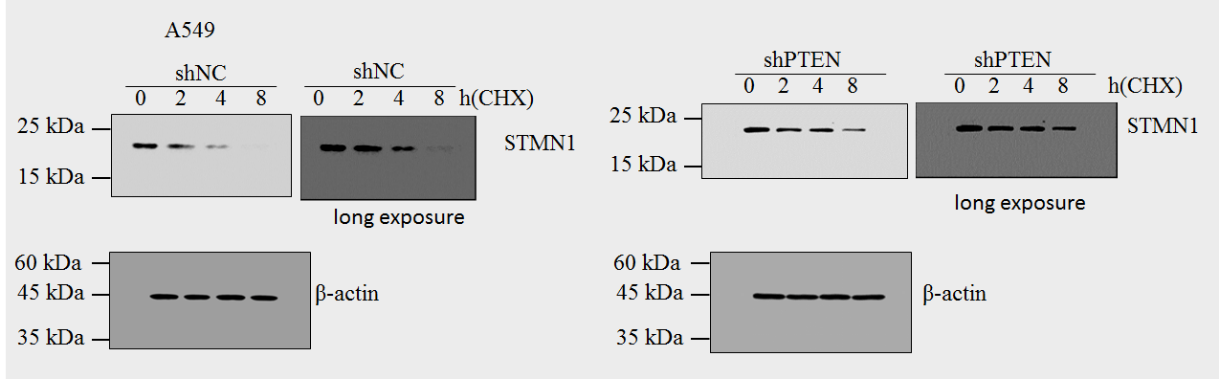


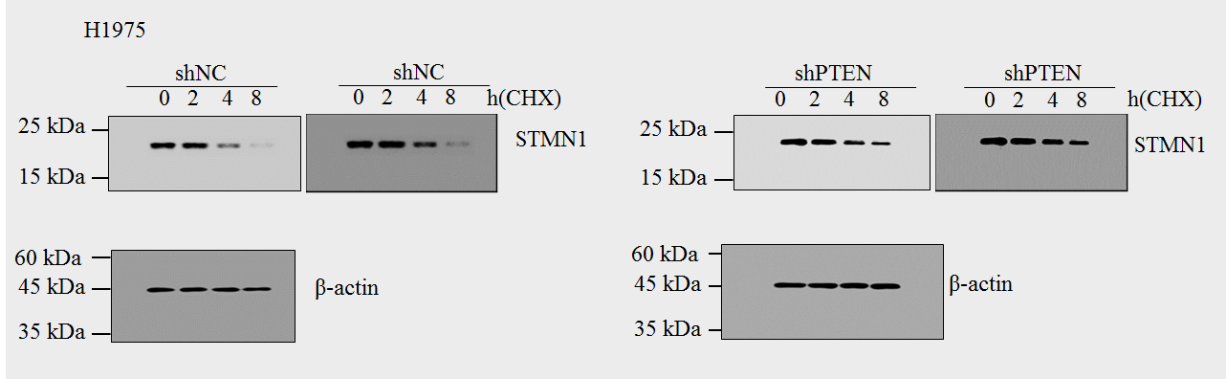


Supplementary figure 9. When STMN1were knocked down and synergized with LY294002 treatment in A549 and H1975 cells, the cell proliferation was examined.


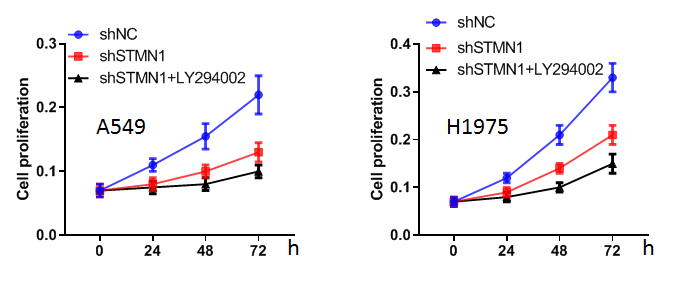


Supplementary Figure 10.


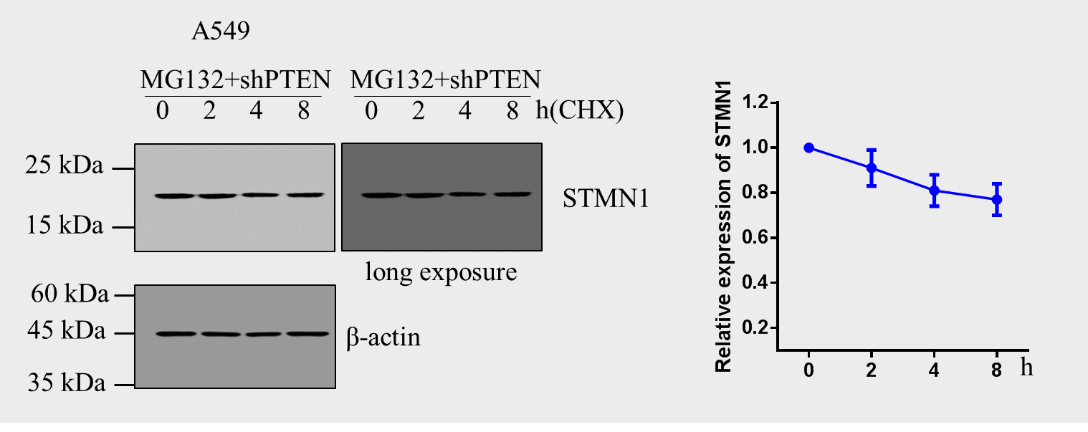


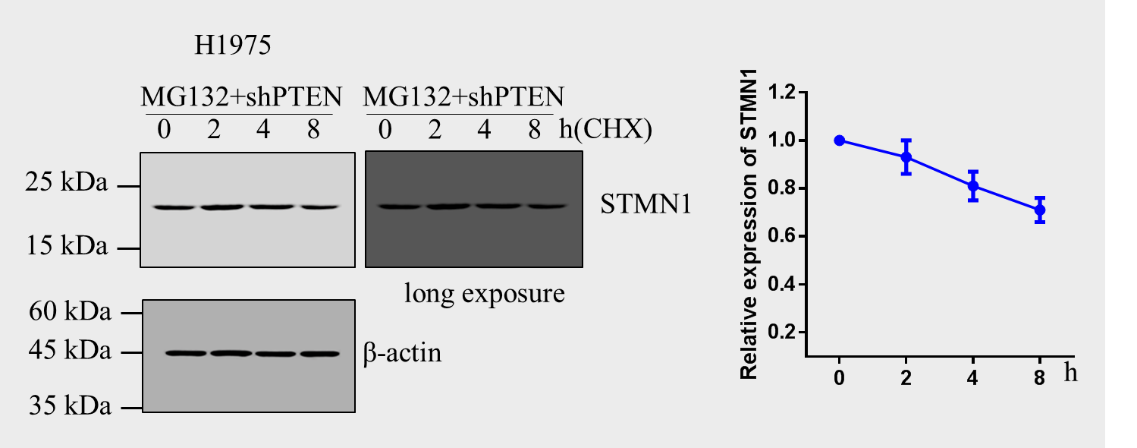


Supplementary figure 11. Original blot data of figure 5a.


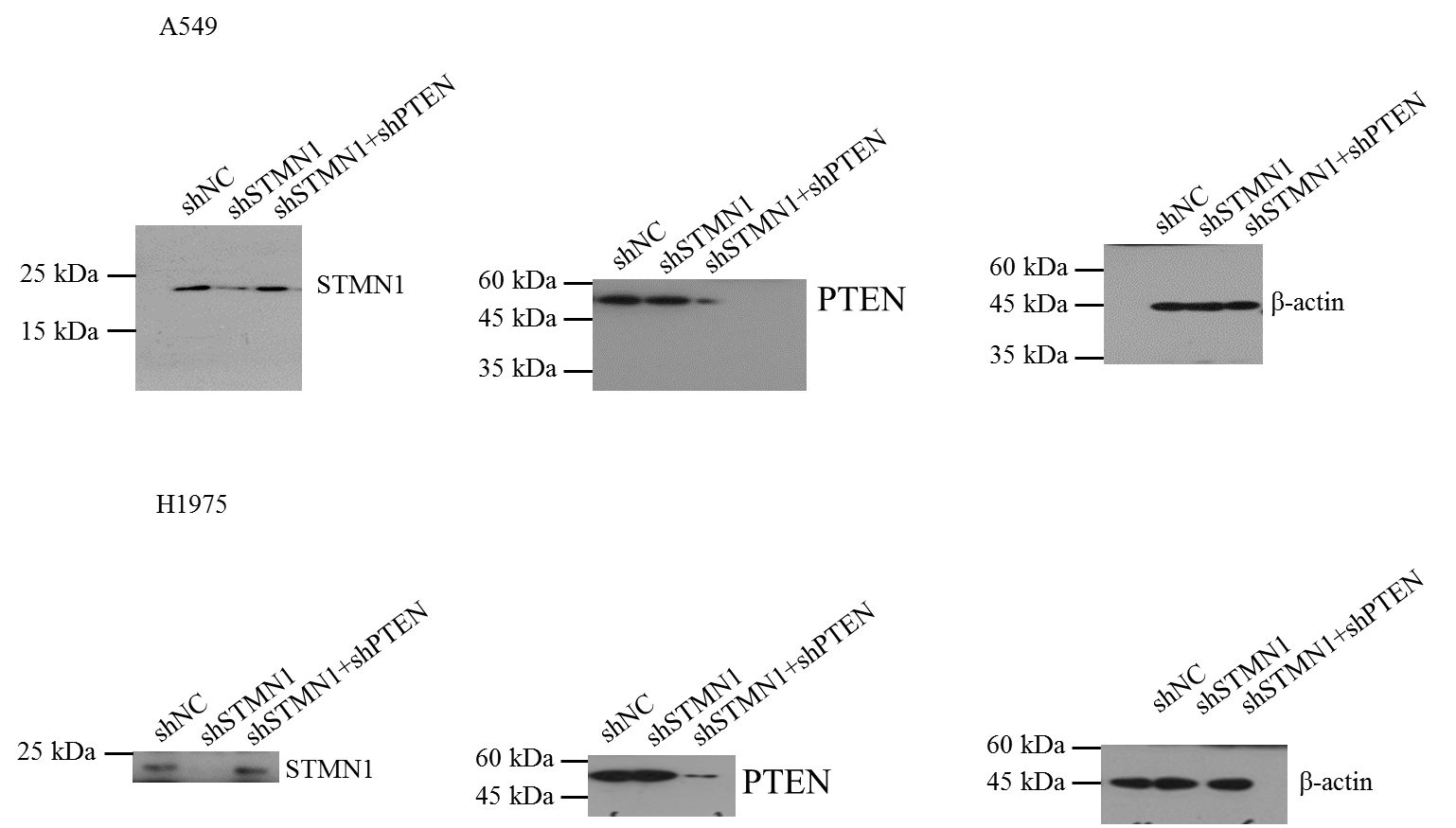

Supplement: Supplementary file 1 — Supplementary Figures. [file 41598_2021_93815_MOESM1_ESM.docx]
